# Definitive irradiation as a first treatment strategy for primary and metastatic sites of newly diagnosed IVB cervical cancer that presented with synchronous oligometastases

**DOI:** 10.1186/s13014-023-02320-6

**Published:** 2023-09-26

**Authors:** Junyun Li, Ying Wang, Lanqing Huo, Xiaodan Huang, Liu Shi, Lin Huang, Kai Chen, Xinping Cao

**Affiliations:** Department of Radiation Oncology, State Key Laboratory of Oncology in South China, Sun Yat-sen University Cancer Center, No. 651 Dongfeng Road East, Guangzhou, 510060 Guangdong P. R. China

**Keywords:** Cervical cancer, Oligometastases, Definitive irradiation

## Abstract

**Background:**

The present study identified survival and progression-free rates and evaluated prognostic factors for IVB stage cervical cancer in patients that presented with synchronous oligometastases (sync-oligometastases) who received definitive irradiation for primary and metastatic sites.

**Methods:**

The study retrospectively included 60 patients with newly diagnosed stage IVB cervical cancer. Patients received definitive radiation for both primary and metastatic sites through Volumetric Modulated Arc Therapy (VMAT) or intensity modulated radiation therapy (IMRT) followed by three dimensional-intracavitary/interstitial brachytherapy at our institution between July 2014 to December 2020. All patients were staged based on the International Federation of Gynecology and Obstetrics (FIGO) 2018 guidelines. Overall survival (OS), progression-free survival (PFS), and patient prognostic factors were analyzed.

**Results:**

The 60 patients who received curative-intent irradiation for primary and metastatic sites showed a 5-year OS rate of 51.4% and a 5-year PFS rate of 25.9%. The median PFS was 52.3 months, and the median OS had not been reached. Lymphatic metastases had a better OS compared with hematogenous metastases (3-year OS rates: 57.2% vs. 20%, p = 0.017). Patients with one metastasis site showed a more favorable prognosis than patients with ≥ 2 metastases sites (3-year OS rates: 60.4% vs. 20.6%, p = 0.003). Patients that presented with tumors larger than 4 cm in diameter before treatment demonstrated a poorer prognosis (5-year OS rates: 41.2% vs. 65.2%, p = 0.029; 5-year PFS rates: 10.4% vs. 53.7%, p = 0.021).

**Conclusion:**

Definitive irradiation for both primary and oligo-metastatic sites for selected IVB patients is a feasible treatment strategy. Metastatic type, number of metastatic sites, and pre-treatment tumor diameter were significant prognostic factors. Neoadjuvant chemotherapy, the lymph nodal metastatic type (supraclavicular or inguinal), and number of lymphatic metastatic sites failed to reach statistical significance as prognostic factors.

## Background

Cervical cancer (CC) ranks as the fourth-leading cause for cancer-related death in females worldwide, with an estimated 260,000 deaths annually [1]. Stage IVB disease accounts for approximately 5% of all cervical neoplasms and the patients’ prognosis is poor. The 5-year overall survival rate for stage IVB patients ranges from 9.9 to 14.6% according to the 2009 International Federation of Gynecology and Obstetrics (FIGO) staging system [2, 3]. No standard treatment strategy for these patients exists because the patients’ state is heterogeneous in nature and ranges from patients with metastasis confined to supraclavicular and/or inguinal lymph nodes to patients with oligo or multiple organ metastases.

Several reports have explored stage IVB cervical cancer, and most used the old FIGO staging system. Patients with only para-aortic lymph node metastases grouped into stage IIIC2 show a significantly higher overall survival using the new staging system (defined as stage IVB in the old staging system). According to the International Journal of Gynecological Cancer, 5-year OS rates in patients with para-aortic lymph node metastases, lymphatic metastases excluding only para-aortic lymph node metastases, and hematogenous metastases were 59.4%, 24.8%, and 6.1%, respectively [4]. Practical Radiation Oncology reported that definitive local therapy was associated with improved OS in patients with newly diagnosed stage IVB cervical cancer (the median OS was 19.2 months in the definitive local therapy group and 10.1 months in the conservative therapy group) [5]. However, the definitive local therapy included chemoradiation therapy and definitive surgery, and the metastatic sites received only systemic chemotherapy with or without palliative local therapy. A previous study showed that whole pelvic radiation and chemotherapy for stage IVB cervical cancer patients showed a significant OS benefit (41.6 months vs. 17.6 months, p < 0.01) [6]. However, the chemotherapy and radiation sequence as well as the dose and type of radiation were not collected.

We analyzed the outcome of newly diagnosed stage IVB cervical cancer patients with sync-oligometastases using a new staging system that excludes para-aortic lymph node metastases. Recently, sync-oligometastases/metachronous oligometastases (meta-oligometastases)/oligo-recurrence has been one of the most important concepts in oncology. Oligometastases refer to cancer patients with 1–5 metastatic or recurrent lesions, and the status of the primary lesions have no restrictions. Oligo-recurrence is cancer patients with 1–5 metastatic or recurrent lesions that could be treated by local therapy, under conditions of controlled primary lesions. Thus, oligo-recurrence refers to oligometastases that are metachronous. Sync-oligometastases have an active primary lesion [7–9]. Niibe et al. showed characteristics for oligo-recurrence cervical cancer patients [10, 11], and our study focused on sync-oligometastases cervical cancer patients. For these patients, both primary and metastatic sites received definitive radiation with or without concurrent chemotherapy. We hypothesized that a definitive treatment strategy for newly diagnosed stage IVB cervical cancer patients demonstrates an OS benefit.

## Methods

The present study retrospectively included 60 patients with newly diagnosed stage IVB cervical cancer. Patients received definitive radiation by Volumetric Modulated Arc Therapy or intensive modulated radiation therapy followed by three dimensional-intracavitary / interstitial brachytherapy at our institution between July 2014 to December 2020. All patients were staged based on the International Federation of Gynecology and Obstetrics (FIGO) 2018 guidelines. Clinical records were reviewed and the data was summarized, including age, histological cancer type, metastasis sites, radiation dose, chemotherapy regimen, and treatment used (Table 1). Distant metastases were confirmed by imaging (computed tomography, magnetic resonance imaging, and positron emission tomography) or histological examination. Percutaneous needle aspiration biopsies were performed in patients with lymphadenopathy in supraclavicular or groin. None of the patients enrolled in the study received prior treatment. The inclusion criteria were as follows: (1) histologically confirmed primary cervical cancer; (2) histologically confirmed squamous cell carcinoma (SCC), adenocarcinoma (AC), or adenosquamous carcinoma; (3) distant lymph node metastases were histological confirmed when confined to supraclavicular and/or inguinal lymph nodes, and/or oligo organs; (4) stage IVB patients that presented with oligo-metastases at initial diagnosis. Oligo-metastases was defined as a limited number of metastatic lesions (typically ≤ 5). Patients with tumors that were not pathologically confirmed, patients with poor performance status, or without follow-up were excluded.

**Table 1:** Patient and treatment characteristics

Patient and treatment characteristics (n = 60)	
**Midian age (years)**	53 (29–84)
≥ 50 years	40 (67%)
<50 years	20 (33%)
**Histology**	
Squamous cell	56 (93%)
Adenocarcinoma	4 (7%)
**Pre-treatment MRI-based maximal diameter (mm)**	43 (10–82)
>40 mm	35 (58%)
≤40 mm	25 (42%)
**Pre-treatment SCC lever (ng/ml)**	17.7 (0.4–70)
**Chemotherapy**	
**Yes**	50 (83%)
Cycles	4 (1–6)
Regimen	Cisplatin Nedaplatin Platin-based doublet therapy
**No**	10 (17%)
**Initial treatment**	
Neoadjuvant chemotherapy + CCRT/RT	31 (52%)
CCRT	14 (23%)
Radiotherapy alone	10 (17%)
CCRT/RT + adjuvant chemotherapy	5 (8%)
**RT dose (Gy)**	
EBRT to pelvis with or without abdomen	45 (45–50)
Brachytherapy to primary lesion (EQD2)	40 (32–70)
EBRT boost to gross node disease	10 (8–14)
EBRT to distant LN	60 (60–74)
EBRT to metastatic organ	60 (60–70)
**Metastatic Sites at Diagnosis**	
Supraclavicular lymph node	45
Inguinal lymph node	16
Axillary lymph node	4
Mediastinal lymph node	1
Lung Parenchymal metastases	3
Bony metastases in pelvis	3
Extrapelvic bony metastases	4
Other (adrenal gland, gluteus maximus)	2
**Number of Metastatic sites at Diagnosis**	
1	43 (72%)
2	14 (23%)
3	2 (3%)
4	1(2%)

Metastatic sites included supraclavicular lymph nodes (45/60 patients, 75%), inguinal lymph nodes (16/60 patients, 26.7%), axillary lymph nodes (4/60 patients, 6.7%), mediastinal lymph nodes (1/60 patients, 1.7%), lung parenchymal (3/60 patients, 5%), bony metastases in pelvis (3/60 patients, 5%), extra-pelvic bony lesions (4/60 patients, 6.7%), and other lesions (2/60 patients, 3.3%). Seventeen patients had more than one metastatic site and 43 patients displayed one metastatic site. A total prescription dose of 45–50 Gy was delivered to the pelvic field (encompassing extended para-aortic fields in most cases) with VMAT/IMRT in 25 fractions at five fractions per week. Lymph node boosts up to 60–74 Gy were delivered to the gross residual mass. Inguinal metastatic lymph nodes, inguinal lymphatic fields, and bony metastases in the pelvis, achieved spontaneous definitive irradiation. High dose rate brachytherapy with iridium-192 was given after the completion of pelvic external beam radiation. A median prescribed dose of 30 Gy was delivered once or twice a week (range 24–36 Gy; 4–6 fractions). Platinum-based regimen (paclitaxel plus cisplatin/paclitaxel plus carboplatin/cisplatin/nedaplatin) were delivered depending on patient tolerance.

Distant metastatic lymph node fields (range 45–50 Gy), distant metastases of lymph nodes (range 60–74 Gy), or extra-pelvis oligo-organs (range 60–74 Gy) were delivered definitive irradiation following radiotherapy of the primary lesion. For primary gross tumors, therapeutic doses were delivered by external beam radiotherapy plus brachytherapy. We used the quadratic linear model [a/β = 10 was adopted for gross tumor volume, and a/β = 3 was adopted for organs at risk (OARs)]. The physical.

dose of image guided adaptive brachytherapy (IGABT) was converted into the bioequivalent dose of 2 Gy. The dose received by 90% of the clinical target volume (HRCTV) of the primary tumor (D90%) and the irradiation dose received by 2 cm^3^ (D 2 cm ^3^) of the high-dose area to the OARs were calculated. Then, the doses of IGABT and pelvic external irradiation were added to obtain the cumulative D90% to the HRCTV and the cumulative D 2 cm ^3^ to the OARs. Dosimetric data for HRCTV, PTV of metastatic sites, and OAR are listed in Table 2. In addition, the platinum-based chemotherapy was given according to patient status. Most patients completed 4–6 cycles of chemotherapy.

**Table 2:** Dosimetric data relating to planning target volumes (PTV) and organs at risk (OAR).

	median Dose (Gy)
HRCTV D_90%_	100 (88–108)
Rectal D2cm^3^	71(67–73)
Intestine D2cm^3^	62(59–66)
bladder D2cm^3^	83(79–88)
PTV bone	72(70–74)
PTV lymph node	60 (60–74)
PTV lung parenchymal	72(70–74)
PTV adrenal gland	60

After completion of treatments, patients were typically followed every three months for 2–3 years, every six months for the next two years, then annually thereafter. Progression-free survival (PFS) was defined as the time from initiation of treatment to the first recurrence of disease, whereas overall survival (OS) was defined as the time from initiation of treatment to death or the last follow-up.

The present study was approved by the Ethics Committee of the Sun Yat-sen University Cancer Center.

Survival curves were estimated using Kaplan-Meier methods, and differences were tested using the log-rank test. P values were calculated using a likelihood ratio test, with values of 0.05 or less considered statistically significant.

## Results

A total of 60 patients received definitive radiation for both primary and metastasis sites. The median age of the patients was 53 years old (range 29–84 years). Most patients were diagnosed with squamous cell carcinoma (93%, n = 54); the remaining patients had adenocarcinoma (7%, n = 4). The median follow-up for these women was 33 months (range 12–90 months).

These 60 patients had a 5-year OS rate of 51.4% and a 5-year PFS rate of 25.9%. The median PFS was 52.3 months, and the median OS has not reached. The mean OS was 40.9 months, and the median PFS was 30 months.

**Fig. 1:**
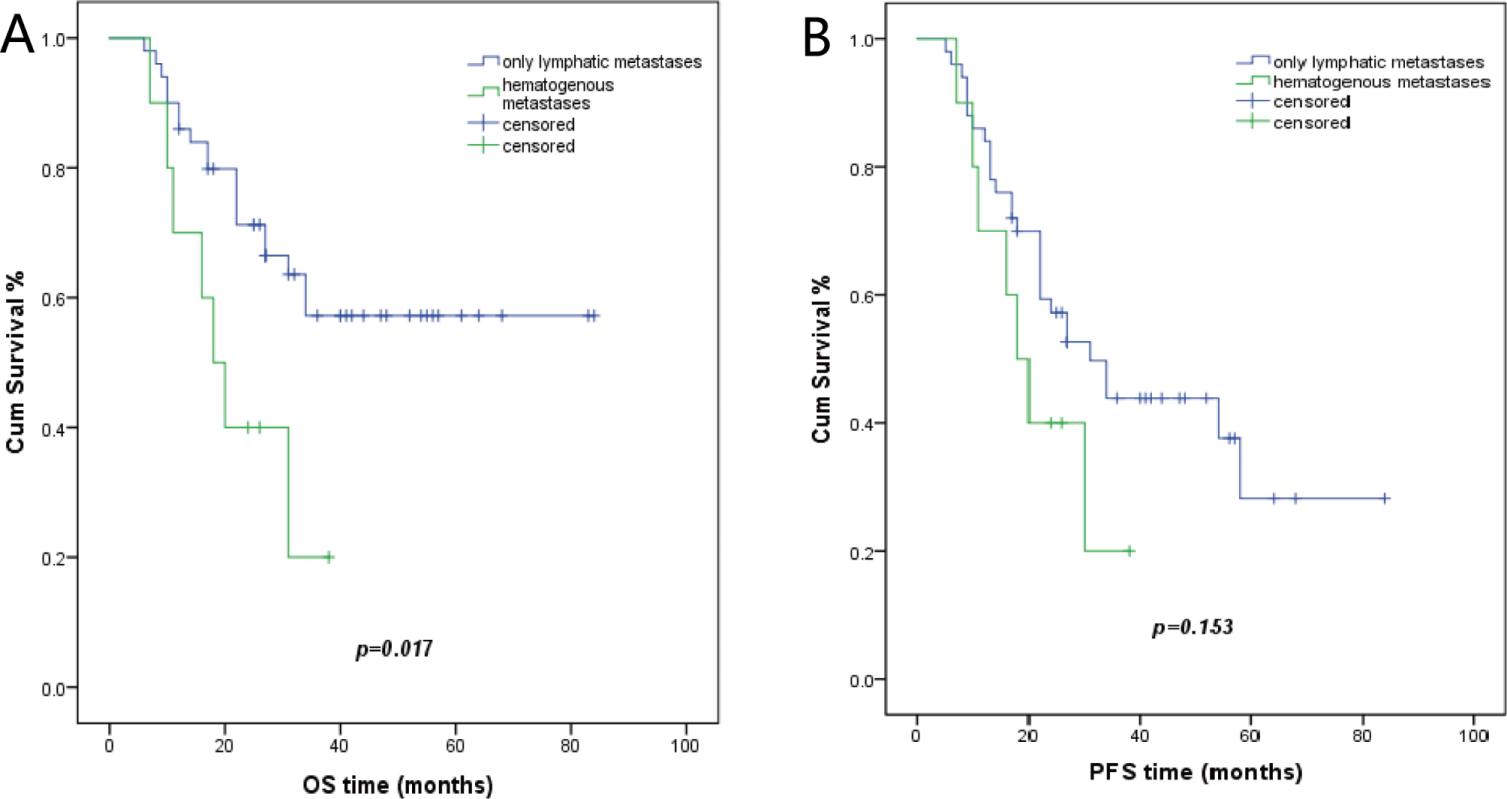
Kaplan Meier plots showing OS and PFS of lymphatic and hematogenous metastatic type **A**, the 3-year OS of patients with only lymphatic metastases or hematogenous metastases [57.2% versus 20% (p = 0.017), respectively]. **B**, the 3-year PFS of patients with only lymphatic metastases or hematogenous metastases [43.9% versus 20% (p = 0.153), respectively]

**Fig. 2:**
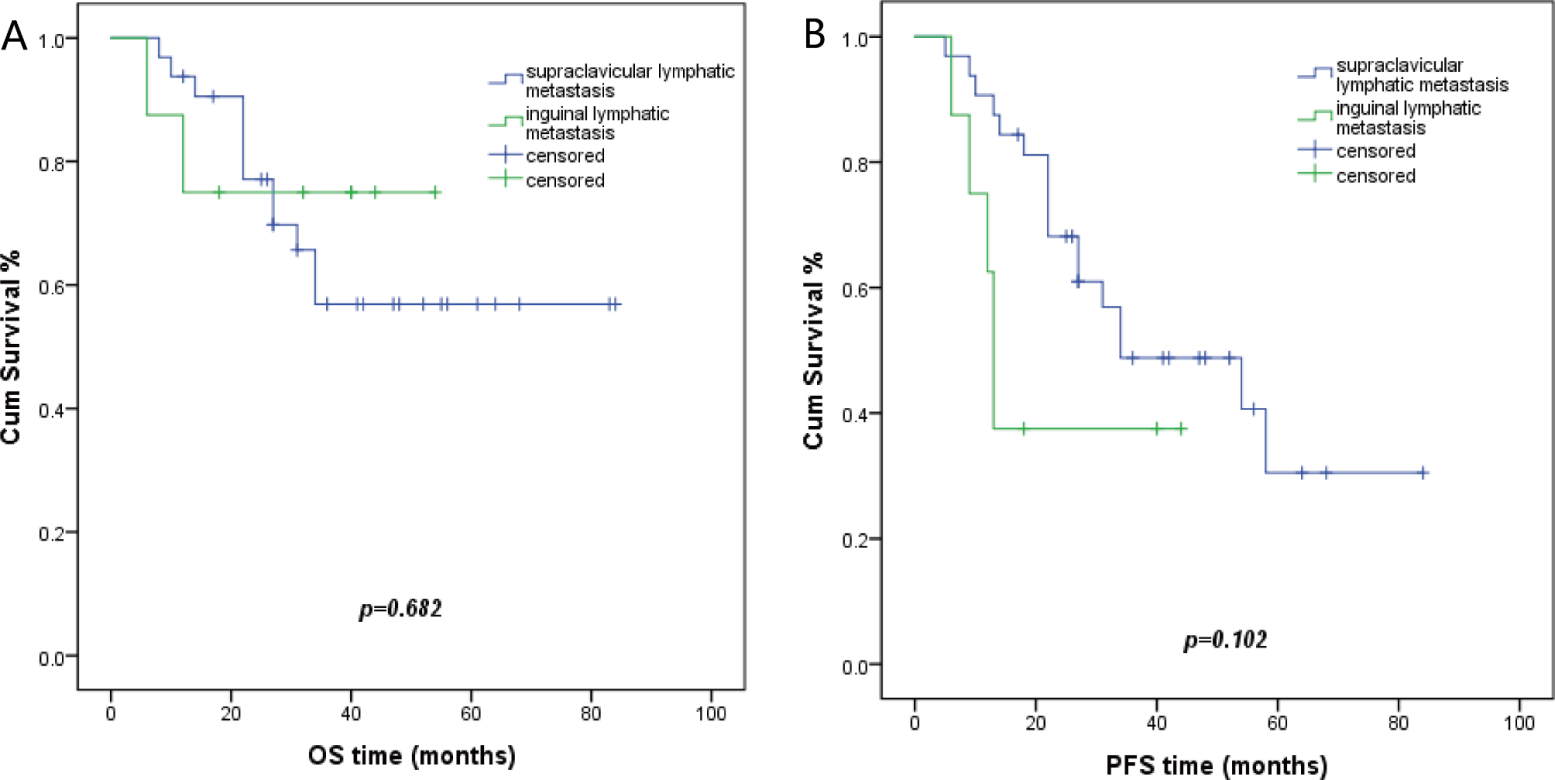
Kaplan Meier plots of OS and PFS in patients with one lymphatic metastasis site. **A**, the 3-year OS for patients with supraclavicular lymph nodes metastasis and patients with inguinal lymph nodes metastasis was 56.9% versus 75% (p = 0.682), respectively. **B**, the 3-year PFS for patients with supraclavicular lymph nodes metastasis and patients with inguinal lymph nodes metastasis was 48.8% versus 37,5% (p = 0.102), respectively

**Fig. 3:**
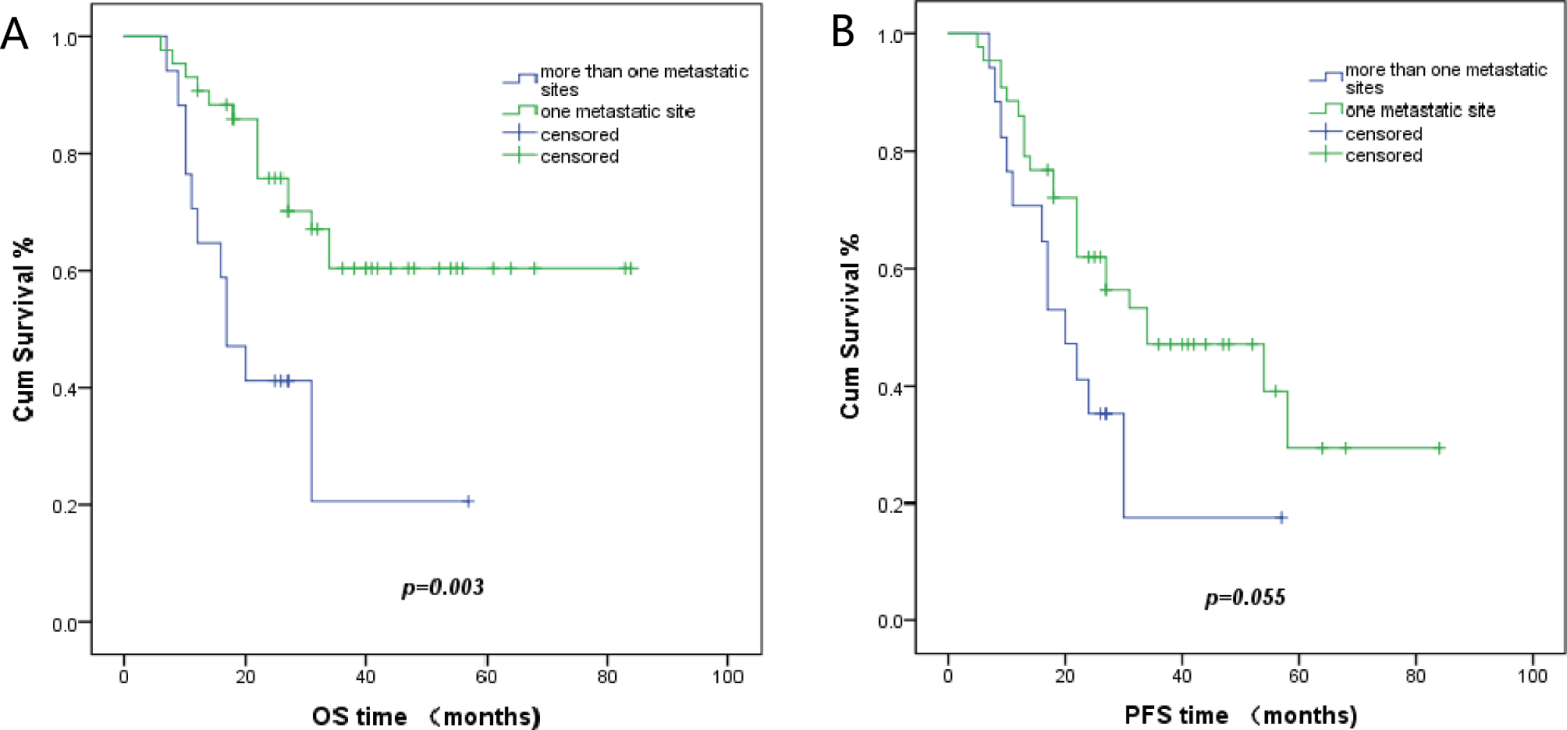
Kaplan Meier plots of OS and PFS in patients with one or > 1 metastatic site. **A**, the 3-year OS of patients with more than one metastatic site and patients with only one metastatic site was 20.6% versus 60.4% (p = 0.003), respectively. **B**, the 3-year PFS of patients with more than one metastatic site and patients with only one metastatic site was 17.6% versus 47% (p = 0.055), respectively

Fifty patients displayed distant (inguinal and/or supraclavicular and/or axillary) lymph node metastases, whereas ten patients had oligo organ metastases with or without distant lymph node metastases. Patients with distant lymph node metastases had a 57.2% 3-year overall survival (OS) rate. Patients with oligo organ metastases demonstrated a 20% 3-year overall survival (OS) rate (p = 0.017). The 3-year progression-free survival (PFS) rates were 43.9% and 20%, respectively (p = 0.153) (Fig. 1). The median PFS was 31 months for patients with distant lymph node metastases and 18 months for patients with oligo organ metastases. Among patients with lymph node metastases, 40 patients had metastasis at one site, whereas 10 patients displayed metastases at two sites. Neither OS or PFS showed statistically significant differences between the metastasis’s groups (Table 3). In the 40 patients with one lymph node metastasis site, 32 patients had supraclavicular lymph node metastasis with a 56.9% 3-year OS rate, eight patients had inguinal lymph nodes metastasis with a 75% 3-year OS rates (p = 0.682) (Fig. 2). The median PFS was 34 months and 13 months, with 3-year PFS rates of 48.8% vs. 37.5%, respectively (p = 0.102). At initial diagnosis, 43 patients showed only one metastatic site, and 17 patients had two or more metastatic sites. The 3-year OS rate was 60.4% in patients with one metastatic site and 20.6% in patients with two or more metastatic sites (p = 0.003). The 3-year PFS rates was 47% in patients with one metastatic site and 17.6% in patients with two or more metastatic sites (p = 0.055) (Fig. 3). The median PFS was 34 months and 20 months, respectively.

**Table 3:** Study characteristics

variable	No.of patients (%)	OS	PFS
		P value	P value
**Age at primary Dx**			
≥ 50 years	40 (67)	0.521	0.938
< 50 years	20 (33)		
**Tumor histology**			
Adenocarcinoma	4 (7)	0.102	0.039
Squamous cell	56 (93)		
**Pre-treatment MRI-based maximal diameter (mm)**			
>40 mm	35(58)	**0.029**	**0.021**
≤40 mm	25(42)		
**type of metastases**			
LN only (lymphatic)	50(83)	**0.017**	0.153
Organ/organ + LN (hematogenous)	10(17)		
**Only one site lymph node metastases**			
Supraclavicular lymph node	32 (80)	0.682	0.102
Inguinal lymph node	8 (20)		
**Number of lymph node metastatic sites (LN only)**			
1	40 (80)	0.146	0.478
2	10 (20)		
**Number of metastatic sites at initial diagnosis**			
1	43 (72)	**0.003**	0.055
≥ 2	17 (28)		
**Neoadjuvant chemotherapy**			
yes	29 (58)	0.261	0.973
no	21 (42)		
**Chemotherapy circles**			
≥ 4	26 (52)	0.364	0.875
< 4	24 (48)		

A total of 50 patients received chemotherapy, and 28 patients had neoadjuvant chemotherapy. Neither OS or PFS showed statistically significant differences in patients with or without adjuvant chemotherapy. Patients that received greater than three chemotherapy rounds (≥ 4) demonstrated a trend in OS benefit, however neither OS nor PFS showed a statistically significance difference.

**Fig. 4:**
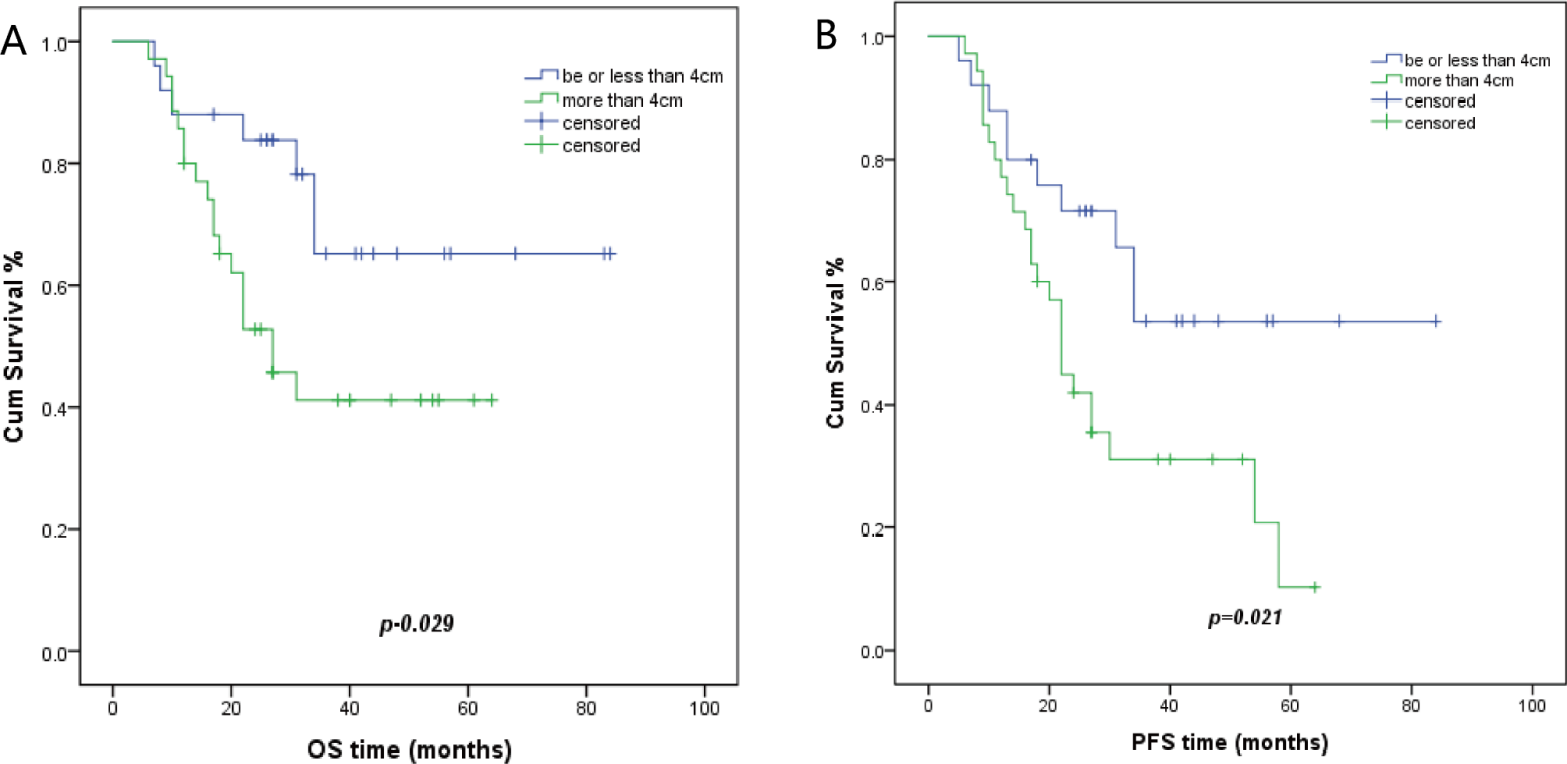
Kaplan Meier plots of OS and PFS of patients with different size tumor diameters before treatment. **A**, the 5-year OS of patients with ≤ 4 cm tumors and > 4 cm tumors was 65.2% versus 41.2% (p = 0.029), respectively. **B**, the 5-year PFS of patients ≤ 4 cm tumors and > 4 cm tumors was 53.7% versus 10.4% (p = 0.021), respectively

Patients with tumor diameter of > 4 cm had a 5-year OS of 41.2%. Patients with a tumor diameter of < 4 cm demonstrated a 5-year OS of 65.2% (p = 0.029). The 5-year PFS was 10.4% and 53.7%, respectively (p = 0.021) (Fig. 4).

Patients with SCC at initial diagnosis showed no statistically significant difference in OS or PFS.

All patients have completed radiation treatment (RT). The median duration of RT (primary site plus metastatic site) was 70 days (range 49–112 days). Simultaneously, the patients received chemotherapy for 4–6 cycles. A total of 24 patients (40%) received 1–3 cycles of radiation. Twenty-six patients (43.3%) completed chemotherapy, and the median chemotherapy cycle number was four (range 1–6 cycles). Acute toxicity was assessed weekly during the course of radiotherapy and 1-month after radiotherapy. Late toxicity was defined as adverse events occurring after 90 days from completion of brachytherapy. For acute toxicity, the incidence of severe (grade 3–4) neutropenia, anemia, and thrombocytopenia was 20%, 38.3%, and 21.7%, respectively. The incidence of severe (grade 3–4) enterocolitis, proctitis, and cystitis was 1.67%, 0%, and 0%, respectively. For late toxicity, the incidence of severe (grade 3–4) enterocolitis, proctitis, and cystitis was 1.67%, 0%, and 0%, respectively (Table 4).

**Table 4:** Acute and late toxicity

		Grade	IMRT/VMAT(n = 60)
**Acute toxicity**	Neutropenia	1–2	26 (43.3%)
		3–4	12 (20%)
	Anemia	1–2	31 (51.7%)
		3–4	23 (38.3%)
	Thrombocytopenia	1–2	5 (8.33%)
		3–4	13 (21.7%)
	Dermatitis	1–2	14 (23.3%)
		3–4	1 (1.67%)
	Nausea	1–2	16 (26.7%)
		3–4	0
	Enterocolitis	1–2	22 (36.7%)
		3–4	1 (1.67%)
	Proctitis	1–2	18 (30%)
		3–4	0
	Cystitis	1–2	11 (18.3%)
		3–4	0
**Late toxicity**	Enterocolitis	1–2	7 (11.7%)
		3–4	1 (1.67%)
	Proctitis	1–2	2 (3.33%)
		3–4	0
	Cystitis	1–2	5 (8.33%)
		3–4	0

## Discussion

The 60 patients in our study who received curative-intent irradiation for primary and metastatic sites showed a 5-year OS rate of 51.4% and a 5-year PFS rate of 25.9%. The median PFS was 52.3 months, and the median OS was not reached. Metastatic type, number of metastatic sites, and tumor diameter before treatment were significant prognostic factors. Neoadjuvant chemotherapy, lymph node metastatic type (supraclavicular or inguinal), or number of lymphatic metastatic sites were not statistically significant prognostic markers.

Patients with stage IVB cervical cancer are usually treated with systemic chemotherapy with or without bevacizumab/PD-1 or palliative radiotherapy for symptom control. A recent phase III trial of bevacizumab for patients with metastatic, persistent, or recurrent cervical cancer showed that chemotherapy plus bevacizumab significantly improved OS compared with chemotherapy alone (16.8 months versus 13.3 months, respectively; p = 0.007) [12]. A previous phase III trial with pembrolizumab for persistent, recurrent, or metastatic cervical cancer assessed the relative benefit of adding pembrolizumab to chemotherapy with or without bevacizumab. In the intention-to-treat population, the median PFS was 10.4 months in the pembrolizumab group and 8.2 months in the placebo group (p < 0.001), and 2-year OS rates were 50.4% and 40.4%, respectively (p < 0.001) [13]. In our study, patients with IVB cervical cancer initially presenting with oligo-metastases had a 5-year OS rate of 51.4% and a 5-year PFS rate of 25.9%. The median PFS of the 60 patients was 52.3 months, and the median OS was not reached. These results suggest that the use of chemotherapy, targeted agents, and immunotherapy may be associated with modest outcome. Definitive irradiation for primary and oligo-metastatic sites showed increased PFS and OS rates compared with chemotherapy plus pembrolizumab with or without bevacizumab. We consider that the superior survival rate and PFS were based on the sufficient dose to the primary and distant gross disease with advanced technologies. Irradiations reduce the risk of further spreading malignant cells and fewer life-threatening complications of gross tumor progression.

Ji-Yoon Kim and his colleague found that curative external beam radiation therapy plus two-dimensional brachytherapy (CCRT) was feasible in patients with stage IVB cervical cancer that presented with para-aortic and left supraclavicular lymph nodal metastases. The 25 patients showed a 49% 3-year OS rate and 33% PFS rate [14]. In our study, 32 patients presented with supraclavicular lymph nodal metastases without additional distant lymph node metastasis. The patients showed 3-year OS and PFS rates of 56.9% and 48.8%, and 5-year OS and PFS rates of 56.9% and 30.5%, respectively. We attributed the superior OS and PFS rates to VMAT/IMRT followed by intracavitary/interstitial three-dimensional brachytherapy. Derks et al. have suggested that the use of three-dimensional brachytherapy showed a trend towards improved local control and overall survival compared with conventional two-dimensional brachytherapy [15]. Since 2006, our institution has used both external beam radiation therapy plus three-dimensional brachytherapy (CT-guided with the uterine tandem and interstitial needles) to treat patients who have undergone definitive radiation.

Previous studies have shown that metastases type was a prognostic factor [16]. The median OS was 14 months, and the 2-year OS rate was 36% in patients with disseminated cervical cancer. A total of 13 patients with supraclavicular lymph node metastases had a 2-year OS rate of 7%. The 2-year OS rate for 17 patients with hematogenous metastases was 75%. Similarly, our study showed a 20% 3-year PFS rate for the 10 patients with hematogenous metastases (oligo-organ with or without metastatic lymph node) and a 43.9% rate for the 50 patients with only lymphatic metastases (p = 0.153). The median PFS was 18 months and 43.9 months, and the 3-year OS rate was 20% and 57.2%, respectively, (p = 0.017). Thus, the type of metastasis was a prognostic factor for prolonged survival.

In our study, there were 40 patients with one distant lymph nodal metastasis site. The 3-year OS in the supraclavicular lymph node group (32 patients) was 56.9%, and 75% in the inguinal lymph node group (8 patients) (p = 0.682). The 3-year PFS was 48.8% and 37.5%, respectively (p = 0.102). Although the supraclavicular metastatic lymph nodes are farther from the primary site compared with the inguinal metastatic lymph nodes, statistical significance between the two sites was not observed.

In addition, we evaluated the efficacy of neoadjuvant chemotherapy. Among patients who received chemotherapy, 28 had neoadjuvant chemotherapy, and no significant difference in OS and PFS was observed between the two chemotherapy groups. An ongoing phase III trial called “INTERLACE” is designed to assess whether the addition of neoadjuvant chemotherapy to standard cisplatin-based chemoradiation improves OS. The findings from INTERLACE may determine if neoadjuvant chemotherapy can result in OS benefit.

In a previous multicenter study, a > 6 cm pre-treatment tumor diameter was an independent prognostic factor for locally advanced cervical cancer [17]. A recent study showed that patients with a pre-treatment tumor size of > 4.4 cm demonstrated worse 5-year OS and 5-year PFS rates (58.3% vs. 84.1% and 52.2 vs. 80.7%, respectively; P = 0.001) [18]. We found that patients with a pre-treatment MRI-based maximal tumor diameter of > 4 cm (35/60, 58%) had reduced 5-year OS and 5-year PFS rates (41.2% vs. 65.2%, P = 0.029, and 10.4% vs. 53.7%, P = 0.021, respectively). Thus, our results were similar to previous studies.

In previous studies, an increased number of treated organs has been associated with shorter OS in oligo-metastatic patients, and metachronous metastases (HR 0.49, p = 0.02) has been shown to be independently related to a favorable OS [19, 20]. In our study, all patients were undergoing metachronous metastases at initial diagnosis. We analyzed the outcomes of patients with single metastatic lesions compared with patients with ≥ 2 metastatic lesions. The single metastatic lesion group (43/60, 72%) showed increased 3-year OS and 3-year PFS rates compared with the ≥ 2 metastatic lesions group (60.4% vs. 20.6%, respectively, P = 0.003; 47% vs. 17.6%, respectively, P = 0.055). The median PFS of these two groups was 34 months and 20 months, respectively. Patients with a single metastatic lesion have a more favorable prognostic value, which is consistent with previous studies.

There were several strengths in our study. The study was the first attempt to deliver definitive irradiation to both primary and metastatic sites for oligo-metastatic cervical cancer patients, and the outcome was feasible. The new staging system was applied, and patients with only para-aortic lymph node metastases were excluded. Supraclavicular and inguinal lymph nodal sites were pathologically confirmed, which results in less false positives. The scale is relatively large, and it seems not to be easy because of the low incidence of this disease situation and little awareness of radiotherapy with curative intent. In addition, all patients were treated with the most advanced techniques, including volumetric modulated arc therapy or intensity-modulated radiation therapy followed by three-dimensional high-dose-rate intracavity combined with interstitial brachytherapy. There were several limitations in our study. The main limitation was that the study was retrospective with its inherent biases. Furthermore, the study was performed at a single-institution using a heterogenous patient population with oligo-metastases. Moreover, the first-line regimen for persistent, metastatic, and recurrent cervical cancer changed during the study period, and our treatment strategies lacked immunotherapy and/or targeted therapy.

Because there is no consensus for the treatment of stage IVB cervical cancer patients, our study provided a new option for a selected group of stage IVB patients. The co-operation of multiple centers can promote the standardized treatment of stage IVB patients with oligo metastatic sites and provide a benefit to these patients.

## Conclusion

The present study demonstrated a new strategy for stage IVB cervical cancer patients with sync-oligometastases. The treatment may be most suitable for a selected group of stage IVB cervical cancer patients. A prospective trial evaluating the efficacy and toxicity of definitive radiation for both primary and metastatic lesions in stage IVB cervical cancer patients with sync-oligometastases is necessary.

## Data Availability

The data presented in this study are available from the corresponding author on reasonable request.

## Abbreviations

VMAT: Volumetric Modulated Arc Therapy
IMRT: intensity modulated radiation therapy
OS: overall survival
PFS: progression-free survival
CC: cervical cancer
FIGO: International Federation of Gynecology and Obstetrics
SCC: squamous cell carcinoma
AC: adenocarcinoma
